# The associations between blood mercury levels and shark meat intake among workers in Gyeongsangbuk-do

**DOI:** 10.1186/s40557-017-0185-9

**Published:** 2017-06-27

**Authors:** Gun Il Park, Young Seok Byun, Man Joong Jeon, Joon Sakong

**Affiliations:** 1 Department of Occupational and Environmental Medicine, Yeungnam University Hospital, 170, Hyeonchung-ro, Nam-gu, Daegu, 42415 Republic of Korea; 2 Department of Preventive Medicine and Public Health, College of Medicine, Yeungnam University, 170, Hyeonchung-ro, Nam-gu, Daegu, 42415 Republic of Korea

**Keywords:** Shark meat, *Jesa*, Blood mercury levels, Dietary exposure, Health examination

## Abstract

**Background:**

Shark meat is used as sacrificial food in Gyeongsangbuk-do, and is a major source of dietary mercury. This study aimed to evaluate the effects of shark meat intake or the ritual of *Jesa* on blood mercury levels within workers living in Gyeongsangbuk-do.

**Methods:**

This study was conducted from September 2016 to October 2016 in two cities of Gyeongsangbuk-do. To compare the differences between urban and rural areas, two workplaces each in Daegu as the urban area and Yeongcheon as the rural area were selected. General characteristics and characteristics related to shark meat consumption of the workers were acquired by personal interviews during their health examination. Blood mercury concentrations were analyzed by the gold amalgamation method using a direct mercury analyzer (DMA-80; Milestone Inc., Shelton, CT, USA).

**Results:**

The shark consumption group had a higher blood mercury concentration than the non-consumption group. The levels of blood mercury increased with the frequency, annual intake, as well as most recent date of shark meat consumption. Moreover, the levels of mercury in blood increased according to the annual frequency of participation in *Jesa* (times per year) and the annual frequency of shark meat consumption during *Jesa* (times per year).

**Conclusions:**

Shark meat intake and the ritual of *Jesa* contributed to an increase in the blood mercury levels of workers in Gyeongsangbuk-do. Therefore, it is necessary to evaluate dietary exposure, occupational as well as other factors that may influence blood mercury concentrations in workers during their health examination, particularly in regions with high mercury exposures.

## Background

Mercury is present in the form of elemental mercury, inorganic mercury and organic mercury. Generally, mercury is considered an environmental pollutant and toxicant, which is not essential for the functioning of human biological processes [1, 2]. Although mercury exists in a liquid state at room temperature, its high vapor pressure is a major contributor to its occupational exposure and environmental contamination, releasing it continuously into the atmosphere [3]. The mercury that is released in the atmosphere from both natural sources and industrial emissions is captured by rainwater, washed into the sea, and biomethylated to organic mercury by marine organisms. This transformed organic mercury accumulates in marine animals and subsequently in humans through the food chain. Exposures to organic mercury can result in insidious damage to the nervous system. Two representative epidemiological outbreaks of environmental contamination in the past were the Minamata disease in Japan, and mercury poisoning in Iraq caused by grain contaminated with organic mercury fumigants [3–5].

Previous studies have focused on the health effects due to acute mercury exposures [6–8]. Recently, concerns on the effects of chronic, low-dose mercury exposure on health have increased. Epidemiological studies conducted in the Faroe Islands and Seychelles have reported the health risks of exposure to low-dose organic mercury [9]. The risk of mercury poisoning by consumption of large fish [10–12], and the correlation of fish intake with higher concentrations of mercury in the blood have been reported previously [13, 14]. Apart from occupational exposures to mercury, generally, the primary source of mercury in humans is the consumption of fish and shellfish [2, 15, 16].

Blood mercury concentrations are reported to be significantly higher in Koreans than those in American, German and Canadian populations [9, 17–19]. Furthermore, blood mercury levels among individuals living in Gyeongsangbuk-do were reported to be significantly higher than among those in other regions within Korea. As a result of the health impact investigation in 2009, shark meat used as sacrificial food in Gyeongsangbuk-do was identified as a major source of mercury exposure [20, 21]. Sacrificial foods are special foods of a region, consumed during rituals dedicated to the ancestors. *Jesa* is a ritual which typically takes place on days when the ancestors died, on the Korean Thanksgiving day i.e. *Chuseok*, and on the Korean New Year’s Day as per the lunar calendar i.e. *Seollal*. After performing *Jesa*, all family members consume sacrificial foods and drinks.

Once absorbed into the human body, mercury is eliminated slowly through the urine, saliva, feces, and sweat. The half-life of inorganic mercury is about 60 days while that of organic mercury is about 70 days. Therefore, peak exposures to both inorganic and organic mercury compounds are hazardous because of their intense effects on the central nervous system [3]. In other words, mercury exposure from both occupational and dietary sources can have additive harmful health effects in humans. There are only a limited number of studies on the association of blood mercury concentrations with shark meat intake or the ritual of *Jesa*. Moreover, even fewer studies have targeted workers in Gyeongsangbuk-do, who are likely to consume large amounts of shark meat. Therefore, this study aimed to evaluate the blood mercury concentrations of workers in Gyeongsangbuk-do. Furthermore, this study evaluated the effects of shark meat intake on blood mercury levels, and the association between blood mercury levels and the ritual of *Jesa*.

## Methods

### Study subjects

To investigate the effects of shark meat intake on blood mercury concentrations, this study was conducted on workers in Gyeongsangbuk-do, where the levels of blood mercury were reported higher than other regions and shark meat was identified as a major source of exposures. Previous study in Gyeongsangbuk-do reported the differences of blood mercury levels and shark meat intake between urban and rural areas [21]. To compare the differences between urban and rural areas, Daegu was selected as the urban area, and Yeongcheon was the rural area. Two workplaces each in Daegu and Yeongcheon were selected, and were manufacturers of automobile parts. However, there were no sources of occupational exposure in these workplaces. This study was conducted from September 2016 to October 2016. The purpose of the study was explained to all subjects during their health examination. Among the 1241 subjects, 753 individuals who agreed to participate in the study were selected as final study participants. All participants gave informed consent to the utilization of personal information and blood samples for this study. The study was approved by the Institutional Review Board of Yeungnam University (No. 7002016-E-2016-001).

### Questionnaire

General characteristics such as age, sex, smoking status, alcohol consumption, and residential area were acquired by personal interviews during the health examination. Additional questionnaires were used for obtaining information related to shark meat consumption such as intake frequency, annual intake (times per year), the date of most recent intake (days ago), the amount of most recent intake, the number of times participated in *Jesa* (per year), and the number of times shark meat consumed in *Jesa* (per year).

### Blood sample collection

Blood sampling was performed during the health examination, and extra blood samples of the participants were used for measuring the total blood mercury concentrations. Blood samples for the analysis were collected directly into heparin-treated vacutainer tubes. All samples were stored in a dry ice box and transferred directly to the laboratory. They were stored at −70 °C until analysis.

### Analysis of total mercury concentrations in blood

Total mercury concentrations in blood were analyzed by the gold amalgamation method using a direct mercury analyzer (DMA-80; Milestone Inc., Shelton, CT, USA). For the accuracy of analysis, the mercury standard solution was made stepwise by 1 to 10 mL according to the required concentrations using a 1000 mg/L standard solution (Sigma-Aldrich Inc., St. Louis, MO, USA). The mercury standard solution was diluted with a 0.001% L-cysteine solution to make 100 mL solution, which was then used for preparing the calibration curves and measuring the absorbance of mercury. A 100 μl of sample from each participant was injected into the sample container of the analyzer. The absorbance of samples was measured at 253.7 nm wavelength, and the concentration of mercury in these samples (mg/mL) was determined using a calibration curve [21]. For the quality control of the laboratory, we participated in German External Quality Assessment Scheme (G-EQUAS) of the Friedrich Alexander University, Erlangen for occupational-medical and environmental medical toxicological analyses.

### Statistical analysis

Statistical analysis was performed using SPSS version 23.0 for windows (IBM corp., Armonk, NY, USA). The general characteristics of study subjects were compared using the chi-square test. The comparison of mean age of the subjects was performed by Student’s *t*-test. The blood mercury concentrations of subjects were log-transformed due to its right skewed distribution and were expressed as the geometric mean. Analysis of covariance (ANCOVA) was used to compare the adjusted means of blood mercury concentrations according to the general characteristics, shark-meat-intake-related characteristics and *Jesa*-related characteristics after controlling confounding variables such as age, sex, smoking status, alcohol consumption and residential area.

## Results

### General characteristics of the study subjects

The general characteristics of the study subjects classified by shark meat consumption are presented in Table 1. Significant differences were observed in the distribution by age groups. The mean age of study subjects was 37.98 ± 7.98 years, and was significantly different between the shark consumption (39.05 ± 7.70) and non-consumption groups (36.89 ± 8.11). Significant differences were also observed in distribution by residential area, and the proportion of participants from the rural area was higher in the shark consumption group than in the non-consumption group. No significant differences were observed in distribution by sex, smoking status and alcohol consumption between the shark consumption and non- consumption groups. No study subjects was occupationally exposed to mercury.

**Table 1:** General characteristics of the study subjects

Characteristics	Total	Shark meat consumption^a^		*p*-value^b^
		Shark consumption	Non-consumption	
Total	753 (100.0)	379 (50.3)	374 (49.7)	
Age (years)				
≤ 29	103 (13.7)	40 (10.6)	63 (16.8)	0.001
30–39	351 (46.6)	163 (43.0)	188 (50.3)	
40–49	231 (30.7)	139 (36.7)	92 (24.6)	
50≤	68 (9.0)	37 (9.8)	31 (8.3)	
Sex				
Men	666 (88.4)	336 (88.7)	330 (88.2)	0.857
Women	87 (11.6)	43 (11.3)	44 (11.8)	
Smoking				
Nonsmokers	242 (32.1)	125 (33.0)	117 (31.3)	0.273
Ex-smokers	155 (20.6)	85 (22.4)	70 (18.7)	
Smokers	356 (47.3)	169 (44.6)	187 (50.0)	
Alcohol				
Nondrinkers	245 (32.5)	115 (30.3)	130 (34.8)	0.196
Drinkers	508 (67.5)	264 (69.7)	244 (65.2)	
Residential area				
Urban	508 (67.5)	189 (49.9)	319 (85.3)	< 0.001
Rural	245 (32.5)	190 (50.1)	55 (14.7)	

Values are expressed as number (percent)

^a^Classified by shark meat consumption

^b^Calculated by chi-square test

### Concentration of blood mercury

The total mercury concentrations in blood of the study subjects depending on general characteristics are presented in Table 2. The blood mercury concentrations were adjusted for age and residential area, because significant differences were observed in their distribution by age and residential area (Table 1). The geometric mean (95% confidential interval) of the total blood mercury concentration in all subjects was 4.11 μg/L (95% CI, 3.94–4.29 μg/L). The shark consumption group had a mean blood mercury concentration of 4.58 μg/L (95% CI, 4.31–4.87 μg/L) which was significantly higher than that in the non-consumption group (3.68 μg/L; 95% CI, 3.47–3.92 μg/L).

**Table 2:** Total blood mercury concentrations depending on general characteristics

Characteristics	Total	Shark meat consumption	
		Shark consumption	Non-consumption
Total	4.11 (3.94–4.29)	4.58 (4.31–4.87)^a^	3.68 (3.47–3.92)^a^
Age (years)			
≤ 29	3.43 (3.06–3.85)	3.35 (2.78–4.04)	3.32 (2.90–3.80)
30–39	4.12 (3.88–4.38)	5.01 (4.57–5.50)	3.44 (3.18–3.72)
40–49	4.35 (4.03–4.70)	5.21 (4.72–5.77)	3.47 (3.10–3.88)
50≤	4.39 (3.82–5.06)	5.68 (4.68–6.90)	3.34 (2.75–4.05)
*p*-value^b^	0.006	< 0.001	0.953
Sex			
Men	4.23 (4.05–4.43)	5.12 (4.80–5.46)	3.50 (3.30–3.71)
Women	3.28 (2.90–3.72)	3.68 (3.06–4.42)	2.87 (2.44–3.37)
*p*-value^b^	< 0.001	0.001	0.022
Smoking			
Nonsmokers	3.68 (3.42–3.97)	4.25 (3.82–4.72)	3.19 (2.88–3.52)
Ex-smokers	4.50 (4.10–4.94)	5.57 (4.90–6.32)	3.60 (3.16–4.10)
smokers	4.26 (4.01–4.53)	5.19 (4.74–5.68)	3.50 (3.24–3.79)
*p*-value^b^	0.001	0.002	0.237
Alcohol			
Nondrinkers	3.79 (3.52–4.08)	4.44 (3.97–4.96)	3.23 (2.94–3.55)
Drinkers	4.28 (4.06–4.50)	5.17 (4.80–5.56)	3.52 (3.29–3.77)
*p*-value^b^	0.008	0.027	0.151
Residential area			
Urban	3.53 (3.35–3.72)	4.32 (3.96–4.72)	3.13 (2.95–3.32)
Rural	5.64 (5.23–6.07)	5.62 (5.16–6.13)	5.70 (4.93–6.59)
*p*-value^b^	< 0.001	< 0.001	< 0.001

Values are adjusted for age and residential area, and expressed as geometric mean (95% confidential interval), unit: μg/L

^a^Significant difference was observed between two groups, *p*-value: < 0.001

^b^Calculated by ANCOVA

The concentrations of blood mercury increased in participants according to their age groups, which showed a peak level in the age group of ≥50 years within all subjects and within the shark consumption group. Moreover, blood mercury concentrations were significantly higher in men than in women within all subjects, the shark consumption group and the non-consumption group. The blood mercury concentrations differed by the smoking status of participants within all subjects and in the shark consumption group. The blood mercury concentrations were significantly higher among alcohol consumers than in non-consumers within all subjects and in the shark consumption group. Blood mercury concentrations significantly differed according to the residential area, with higher concentrations in participants of the rural area than those of the urban area. The geometric mean of blood mercury concentrations in the participants of urban area was 3.53 μg/L, and the geometric mean of blood mercury concentrations in the participants of rural area was 5.64 μg/L.

Table 3 shows blood mercury concentrations of the subjects classified by characteristics related to shark-meat consumption. These blood mercury concentrations were adjusted for age, sex, smoking status, alcohol consumption, and residential area, because significant differences were observed in total blood mercury concentrations according to these general characteristics. Intake frequency, annual intake (times per year) and the date of most recent intake (days ago) were significantly associated with the blood mercury concentrations. However, the amount of most recent intake was not significantly associated with blood mercury concentrations.

**Table 3:** Total blood mercury concentrations by characteristics related to shark-meat consumption

Characteristics	Number (%)	Concentrations^a^	*p*-value^b^
Intake frequency			
Rarely	374 (49.7)	3.70 (3.48–3.93)	<0.001
Only during *Jesa*	365 (48.5)	4.52 (4.25–4.81)	
During and besides *Jesa*	14 (1.9)	5.95 (4.37–8.09)	
Annual intake (times per year)			
0	374 (49.7)	3.71 (3.49–3.94)	<0.001
1–2	212 (28.2)	4.29 (3.97–4.65)	
3–4	103 (13.7)	4.61 (4.12–5.17)	
5≤	64 (8.5)	5.43 (4.71–6.26)	
Date of most recent intake (days ago)			
≤ 7	11 (1.5)	7.33 (5.15–10.43)	0.015
8–30	24 (3.2)	4.29 (3.35–5.50)	
31≤	173 (23.0)	4.12 (3.70–4.59)	
rarely	545 (72.4)	4.06 (3.84–4.28)	
Amount of most recent intake (g)			
rarely	545 (72.4)	4.06 (3.84–4.29)	0.457
≤ 25	66 (8.8)	3.92 (3.35–4.57)	
26–50	86 (11.4)	4.37 (3.80–5.02)	
51≤	56 (7.4)	4.53 (3.82–5.38)	

^a^Adjusted for age, sex, smoking status, alcohol consumption and residential area, and expressed as geometric mean (95% confidential interval), unit: μg/L

^b^Calculated by ANCOVA

Blood mercury concentrations of the subjects by *Jesa-*related characteristics are presented in Table 4. These concentrations were adjusted for age, sex, smoking status, alcohol consumption, and residential area, because significant differences were observed in total blood mercury concentrations according to these general characteristics. Our results show that the levels of blood mercury increased with the frequency of *Jesa* (times per year) and shark meat usage in *Jesa* (times per year) showing a significant association with these characteristics.

**Table 4:** Total blood mercury concentrations by *Jesa-*related characteristics

Characteristics	Total		Shark consumption		Non-consumption	
	Number (%)	Concentrations^a^	Number (%)	Concentrations^a^	Number (%)	Concentrations^a^
Frequency of *Jesa* ^b^						
0	264 (35.1)	3.31 (3.07–3.56)	1 (0.3)	4.18 (1.29–13.54)	263 (70.3)	3.13 (2.93–3.35)
1–2	194 (25.8)	4.20 (3.87–4.55)	162 (42.7)	4.41 (4.03–4.84)	32 (8.6)	4.16 (3.43–5.05)
3–4	190 (25.2)	4.91 (4.52–5.34)	137 (36.1)	5.18 (4.69–5.73)	53 (14.2)	4.53 (3.90–5.27)
5≤	105 (13.9)	4.96 (4.45–5.53)	79 (20.8)	5.70 (5.01–6.50)	26 (7.0)	3.69 (3.00–4.54)
*p*-value^d^		< 0.001		0.010		< 0.001
Shark meat usage in *Jesa* ^c^						
0	310 (41.2)	3.67 (3.43–3.92)	3 (0.8)	4.71 (2.40–9.22)	307 (82.1)	3.38 (3.18–3.59)
1–2	198 (26.3)	4.09 (3.77–4.43)	178 (47.0)	4.41 (4.04–4.82)	20 (5.3)	4.07 (3.20–5.18)
3–4	153 (20.3)	4.77 (4.34–5.23)	126 (33.2)	5.36 (4.83–5.95)	27 (7.2)	3.52 (2.86–4.33)
5≤	92 (12.2)	4.79 (4.25–5.39)	72 (19.0)	5.63 (4.91–6.46)	20 (5.3)	3.31 (2.60–4.20)
*p*-value^d^		< 0.001		0.001		0.508

^a^Adjusted for age, sex, smoking status, alcohol consumption and residential area, and expressed as geometric mean (95% confidential interval), unit: μg/L

^b^Number of times participated in *Jesa* (per year)

^c^Number of times shark meat consumed in *Jesa* (per year)

^d^Calculated by ANCOVA

*GM* geometric mean

*CI* confidential interval

## Discussion

Blood mercury levels among individuals in Gyeongsangbuk-do were reported to be significantly higher than in other regions within Korea, and shark meat usage in *Jesa* was identified as a major source of exposures [21]. However, only a limited number of studies have evaluated the blood mercury concentrations of workers in Gyeongsangbuk-do, who are likely to consume large amounts of shark meat. In this study, we provided representative blood mercury levels of these workers, and confirmed that shark meat intake along with the rituals of *Jesa* contributed to the increase in blood mercury levels of these workers, which is similar to the results of a previous study, the health impact investigation (2009) [21].

According to the Korean National Health and Nutrition Examination Survey (KNHANES), the geometric mean of total mercury concentrations in blood was 4.28 μg/L in 2009 and 3.64 μg/L in 2010. Similarly, the geometric mean of total mercury concentrations in blood measured by the 2009 and 2010 Korean National Environmental Health Survey (KNEHS) were 3.93 μg/L/L and 2.88 μg/L, respectively. In this study, the total mercury levels in blood of the subjects are similar to or slightly higher than the above results, while they are slightly lower than that reported in the previous studies conducted in Gyeongsangbuk-do [9, 21]. The geometric means of total mercury concentrations in blood in our study were: 4.11 μg/L (all subjects), 4.58 μg/L (shark consumption group) and 3.68 μg/L (non-consumption group). The mean blood mercury concentration in the shark consumption group was significantly higher than that in the non-consumption group.

The blood mercury levels in 36.3% of all subjects (273/753) were >5 μg/L (HBM I), which is the limit level for no adverse health effects, while the blood mercury levels in 2.1% of all subjects were >15 μg/L (HBM II), which could increase adverse health effects. On the other hand, 0.7% of the German population are estimated to exceed the levels of HBM I, and none are estimated to exceed HBM II [22]. Consequently, the blood mercury levels in Koreans is significantly higher than that in the populations of other countries such as U.S. (0.86 μg/L), Germany (0.58 μg/L), and Canada (0.76 μg/L) [9, 17–19], which means that the level of mercury exposure in Korea is significantly higher than in other countries. In general, mercury exposure is mostly caused by consumption of fish and shellfish [2, 15, 16]. In Korean population, the estimated daily intake of mercury from shark consumption was higher than the guidelines established by international regulatory authorities [23]. Moreover, the use of shark meat as sacrificial food was reported to be a major source of mercury exposure in Gyeongsangbuk-do [21]. In this study, we confirmed that the levels of blood mercury increased with intake frequency, annual intake (times per year), the frequency of *Jesa* (times per year) and shark meat usage in *Jesa* (times per year). The concentration of blood mercury showed a peak level when the annual intake (times per year) was ≥5 (5.43 μg/L) within all subjects. Within the shark consumption group, it was also observed when the frequency of *Jesa* (times per year) was ≥5 (5.70 μg/L). Both figures exceed HBM I, which suggests that a caution for adverse health effects is required.

No subjects in this study were occupationally exposed to mercury. However, in other workplaces such as thermometer manufacturing plants or fluorescent lamp manufacturing plants, workers may be occupationally exposed to mercury [9]. Essentially, mercury poisoning cases were also reported in the process of demolition work of fluorescent lamp manufacturing plant in Korea in 2015. If these workers are exposed to several dietary sources of mercury like fish or shellfish, mercury levels in their bodies could increase substantially leading to more intense health consequences [3]. Meanwhile, previous studies have mainly focused on the health effects due to acute mercury exposure and the occupational mercury sources among workers [6–8]. However, considering the effects of dietary sources on mercury levels, detailed information on dietary sources is needed during the health examinations, particularly, among the workers in Gyeongsangbuk-do, who are likely to consume a lot of shark meat.

This study has several limitations. The general population or vulnerable subpopulations such as children and pregnant women are mostly excluded, and the workers who agreed to participate in the study during their health examination were selected as the study subjects. Other dietary factors contributing to increase in the blood mercury levels were not considered. Although there were no self-reported sources of occupational mercury exposure in the study subjects, mercury levels in the urine were not analyzed. Urinary mercury levels reflect the exposure to elemental and inorganic mercury, while blood mercury levels reflect the exposure to organic mercury through diet [24, 25]. Therefore, in future studies, various dietary factors contributing to mercury exposure should be investigated in addition to shark meat. In workers occupationally exposed to mercury, a urinary mercury test, which evaluates the levels of inorganic mercury, should also be included. Furthermore, in regions with high mercury exposures like Daegu and Gyeongsangbuk-do, it is necessary to evaluate dietary, occupational, and other factors that may influence the mercury concentration in workers during their health examinations.

## Conclusions

This study investigated the effects of shark meat intake on blood mercury levels, and the association between blood mercury levels and the ritual of *Jesa*. Shark meat intake along with the rituals of *Jesa* contributed to the increase in blood mercury levels of workers in Gyeongsangbuk-do, which is similar to the results of a previous study, the health impact investigation (2009). Blood mercury levels among individuals in Gyeongsangbuk-do were reported to be significantly higher than in other regions within Korea, and shark meat usage in *Jesa* was identified as a major source of exposures. In regions like Daegu and Gyeongsangbuk-do, it is necessary to evaluate dietary and occupational factors that may influence the mercury concentration in workers during their health examinations.

## Abbreviations

CI: Confidential interval
GM: Geometric mean
HBM: Human biomonitoring
KNEHS: Korean National Environmental Health Survey
KNHANES: Korean National Health and Nutrition Examination Survey

## References

[1] World Health Organization (1991). Environmental health criteria 118: inorganic mercury.

[2] Agency for Toxic Substances and Disease Registry (1999). Toxicological profile for mercury (update).

[3] Joseph L, Robert H (2014). Current occupational and environmental medicine.

[4] Kurland LT, Faro SN, Siedler H (1960). Minamata disease. The outbreak of a neurologic disorder in Minamata, Japan, and its relationship to the ingestion of seafood contaminated by mercuric compounds. World Neurol.

[5] Bakir F, Damluji SF, Amin-Zaki L, Murtadha M, Khalidi A, Al-Rawi NY (1973). Methylmercury poisoning in Iraq. Science.

[6] Cha CW, Kim KJ, Yum YT (1992). A study on the mercury contamination sources and risk for occupational mercury poisoning of mercury exposed workers in Korea. Korean J Occup Environ Med..

[7] Kim CY, Kim KJ, Hong D (1993). A study on the blood zinc-protoporphyrin and serum cholinesterase activity of workers exposed to mercury vapor. Korean Ind Hyg Assoc J.

[8] Shin SR, Han AL (2012). Improved chronic fatigue symptoms after removal of mercury in patient with increased mercury concentration in hair toxic mineral assay: a case. Korean J Fam Med.

[9] Ye BJ, Kim BG, Jeon MJ, Kim SY, Kim HC, Jang TW (2016). Evaluation of mercury exposure level, clinical diagnosis and treatment for mercury intoxication. Ann Occup Environ Med.

[10] Hightower JM, Moore D (2003). Mercury levels in high-end consumers of fish. Environ Health Perspect.

[11] Knobeloch L, Steenport D, Schrank C, Anderson H (2006). Methylmercury exposure in Wisconsin: a case study series. Environ Res.

[12] You CH, Kim BG, Kim YM, Lee SA, Kim RB, Seo JW (2014). Relationship between dietary mercury intake and blood mercury level in Korea. J Korean Med Sci.

[13] Turner MD, Marsh DO, Smith JC, Inglis JB, Clarkson TW, Rubio CE (1980). Methylmercury in populations eating large quantities of marine fish. Arch Envion Health.

[14] Kim CW, Kim YW, Chae CH, Son JS, Park SH, Koh JC (2010). The effects of the frequency of fish consumption on the blood mercury levels in Koreans. Korean J Occup Environ Med.

[15] Auger N, Kofman O, Kosatsky T, Armstrong B (2005). Low-level methylmercury exposure as a risk factor for neurologic abnormalities in adults. Neurotoxicology.

[16] Clarkson TW, Vyas JB, Ballatori N (2007). Mechanisms of mercury disposition in the body. Am J Ind Med.

[17] U.S. Department of Health and Human Services, Centers for Disease Control and Prevention (2012). Fourth national report on human exposure to environmental chemicals, updated tables.

[18] Becker K, Kaus S, Krause C, Lepom P, Schulz C, Seiwert M (2002). German environmental Survey 1998 (GerES III): environmental pollutants in blood of the German population. Int J Hyg Environ Health.

[19] Wong SL, Lye EJ (2008). Lead, mercury and cadmium levels in Canadians. Health Rep.

[20] Cho S, Jacobs DR, Park K (2014). Population correlates of circulating mercury levels in Korean adults: the Korea National Health and Nutrition examination Survey IV. BMC Public Health.

[21] National Institute of Environmental Research (2011). Assessment of mercury exposure and health in Daegu and Gyeongsangbuk-do (I).

[22] Schulz C, Angerer J, Ewers U, Kolossa-Gehring M (2007). The German human biomonitoring commission. Int J Hyg Environ Health.

[23] Kim SJ, Lee HK, Badejo AC, Lee WC, Moon HB (2016). Species-specific accumulation of methyl and total mercury in sharks from offshore and coastal waters of Korea. Mar Pollut Bull.

[24] Schober SE, Sinks TH, Jones RL, Bolger PM, McDowell M, Osterloh J (2003). Blood mercury levels in US children and women of childbearing age, 1999–2000. JAMA.

[25] Caldwell KL, Mortensen ME, Jones RL, Caudill SP, Osterloh JD (2009). Total blood mercury concentrations in the U.S. population: 1999–2006. Int J Hyg Environ Health.

